# Identification of acquired mutations by whole-genome sequencing in GATA-2 deficiency evolving into myelodysplasia and acute leukemia

**DOI:** 10.1007/s00277-014-2090-4

**Published:** 2014-04-30

**Authors:** Tohru Fujiwara, Noriko Fukuhara, Ryo Funayama, Naoki Nariai, Mayumi Kamata, Takeshi Nagashima, Kaname Kojima, Yasushi Onishi, Yoji Sasahara, Kenichi Ishizawa, Masao Nagasaki, Keiko Nakayama, Hideo Harigae

**Affiliations:** 1 Department of Hematology and Rheumatology, Tohoku University Graduate School, 2-1 Seiryo-cho, Aoba-ku, Sendai, 980-8575 Japan; 2 Molecular Hematology/Oncology, Tohoku University Graduate School, Sendai, Japan; 3 Department of Cell Proliferation, United Center for Advanced Research and Translational Medicine, Tohoku University Graduate School, Sendai, Japan; 4 Department of Integrative Genomics, Tohoku Medical Megabank Organization, Tohoku University, Sendai, Japan; 5 Department of Pediatrics, Tohoku University Graduate School, Sendai, Japan; 6 Clinical Research, Innovation and Education Center, Tohoku University Hospital, Sendai, Japan

**Keywords:** GATA-2, GATA-2 deficiency, MonoMAC, Myelodysplastic syndrome, Whole-genome sequencing, EZH2, GATA-1

## Abstract

Heterozygous *GATA*-*2* germline mutations are associated with overlapping clinical manifestations termed GATA-2 deficiency, characterized by immunodeficiency and predisposition to myelodysplastic syndrome (MDS) and acute myeloid leukemia (AML). However, there is considerable clinical heterogeneity among patients, and the molecular basis for the evolution of immunodeficiency into MDS/AML remains unknown. Thus, we conducted whole-genome sequencing on a patient with a germline GATA-2 heterozygous mutation (c. 988 C > T; p. R330X), who had a history suggestive of immunodeficiency and evolved into MDS/AML. Analysis was conducted with DNA samples from leukocytes for immunodeficiency, bone marrow mononuclear cells for MDS and bone marrow-derived mesenchymal stem cells. Whereas we did not identify a candidate genomic deletion that may contribute to the evolution into MDS, a total of 280 MDS-specific nonsynonymous single nucleotide variants were identified. By narrowing down with the single nucleotide polymorphism database, the functional missense database, and NCBI information, we finally identified three candidate mutations for *EZH2*, *HECW2* and *GATA*-*1*, which may contribute to the evolution of the disease.

**Electronic supplementary material:**

The online version of this article (doi:10.1007/s00277-014-2090-4) contains supplementary material, which is available to authorized users.

## Introduction

GATA-2 is a zinc finger transcription factor that plays crucial roles in hematopoiesis, as well as vascular, lymphatic, and neural development [1]. Recently, heterozygous *GATA*-*2* germline mutations, both inherited and de novo, were reported to cause three overlapping clinical entities, characterized by a predisposition to myelodysplastic syndrome (MDS) and acute myeloid leukemia (AML): (1) familial MDS/AML, (2) Emberger syndrome and (3) an immunodeficiency termed monocytopenia characterized by mycobacterium avium complex (MonoMAC)/dendritic cell, monocyte, B- and NK-lymphoid deficiency (DCML) [2–4]. All these conditions are generally named “GATA-2 deficiency” syndrome.

Nearly half of the individuals presenting with GATA-2 mutations will eventually develop MDS/AML, associated with fibrosis and megakaryocyte dysplasia. In contrast, many patients gradually develop DCML before MDS/AML, initially detected as mild chronic neutropenia, monocytopenia and/or NK deficiency [2–4]. Therefore, there is considerable clinical heterogeneity among patients with GATA-2 deficiency, although all these conditions predominantly affect the hematologic and immune systems. In addition, the rate of evolution of the disease into MDS/AML appears to be rapid, with varying MDS and AML phenotypes and variable cytogenetic abnormalities [5, 6]. Therefore, secondary genetic events may explain the clinical heterogeneity among cases of GATA-2 deficiency. In this regard, the most commonly associated cytogenetic finding is monosomy 7 and additional acquired mutations, such as those in *ASXL1* [6]. However, the molecular basis for the evolution of GATA-2 deficiency into MDS/AML has not been elucidated, which affects our ability of early detection and treatment of the disease.

Whole-genome sequencing has several advantages over candidate gene sequencing. It provides a comprehensive and nonbiased approach to mutation detection. More importantly, whole-genome paired-end sequencing is able to detect structural variants (SV; e.g., deletions, amplifications, inversions and translocations). Therefore, to investigate the genetic changes associated with the evolution of GATA-2 deficiency into MDS/AML, we performed whole-genome sequencing of MDS sample, which was compared with matched samples from nail, leukocyte at immunodeficiency, and bone marrow-derived mesenchymal stem cells (BM-MSCs).

## Patient and methods

### Study design and clinical samples

All clinical samples were obtained from a single patient referred to our department for pancytopenia and emergence of myeloblasts in the peripheral blood. They included nails, peripheral leukocytes at immunodeficiency (MonoMAC), and bone marrow mononuclear cells for MDS (MDS). The patient signed an informed consent before sample collection, and all ethical considerations were followed according to the Declaration of Helsinki. This study was approved by the ethical committee of the Tohoku University Graduate School of Medicine.

### Cell culture

Cells were grown in a humidified incubator at 37 °C with 5 % carbon dioxide. Human K562 erythroleukemia cell lines were maintained in Roswell Park Memorial Institute (RPMI-1640) medium containing 10 % fetal bovine serum (Biowest) and 1 % penicillin–streptomycin (Sigma). PLAT-GP Packaging Cell Lines (Cell Biolabs) was maintained in Dulbecco’s modified Eagle medium (DMEM) containing 10 % fetal bovine serum (Biowest) and 1 % penicillin–streptomycin (Sigma).

### Gene transfer and vectors

*GATA*-*2* mRNA was cloned into pBABE-puro vector (Addgene Plasmid 1764) [7], and a single mutation was introduced with QuikChange Site-Directed Mutagenesis Kit (Agilent). The retroviral vector encoding human GATA-2 and the env (envelope glycoprotein) gene from the vesicular stomatitis virus (VSV-G) were co-transfected into PLAT-GP cells with FuGene HD (Promega). Seventy-two hours after transfection, the viral supernatant was used for infection. After spin infection into CD34-positive cells at 3,400 rpm for 2 h, the cells were cultured containing 1 μg/mL Puromycin (Sigma) for the selection of the transduced cells.

### Quantitative ChIP analysis

Real-time-PCR-based quantitative chromatin immunoprecipitation (ChIP) analysis was conducted essentially as described [8]. Cells were crosslinked with 1 % formaldehyde for 10 min at room temperature. The nuclei lysate was sonicated to reduce DNA length using Sonifier (Branson). The protein-DNA complexes were immunoprecipitated by specific antibody and Protein A Sepharose (Sigma). Immunoprecipitated DNA fragments were quantified by real-time PCR to amplify regions of 75–150 bp overlapping with the appropriate motif. Product was measured by SYBR Green fluorescence in 20-μL reactions, and the amount of product was determined relative to a standard curve generated from titration of input chromatin. Analysis of post-amplification dissociation curves showed that primer pairs generated single products.

### Primers

Primers used in the study were listed in Table 1.

**Table 1:** Oligonucleotide primers

Designation	Forward and reverse sequences(5′-3′)
Primers used for GATA-2 sequencing	
*GATA*-*2* exon 2	GTTTTGAGCCTTGGGCTTT
	CAATTTTTCAGCAGCTCGATT
*GATA*-*2* exon 3	GGAGTCGTGATCTCAATGTCTG
	ATCTGCTGGGGGCTATTAGAG
*GATA*-*2* exon 4	ACTCCCTCCCGAGAACTTG
	CGTCTGCATTTGAAGGAGTTT
*GATA*-*2* exon 5	GAGATTTAGCCCTCCTTGACTG
	AGCACAAAGCGCAGAGGT
*GATA*-*2* exon 6	GAAGGTCGGGCACAATTC
	ACAGGTGCCATGTGTCCA
Primers used for validation sequencing	
*EZH2*	CATCAAAAGTAACACATGGAAACC
	GCTGCTTTAAAACATAATTCCACA
*HECW2*	GTCCATATCCTACCTCCAGTAGC
	GACAGCTCCTGCAATGAGAGT
*GATA*-*1*	TAGACCTTGGGCAGCTCCT
	CCTTGGTAGAGATGGGCAGTA
Primers used for quantitative ChIP	
*GATA*-*2* −2.4 kb	GTGGAGCTCTAGGGTACCATTT
	TGAGGACACCTCATTAGAGCAG
*GATA*-*2* −3.5 kb	GTCCGGGGTAATTTTTCATCT
	GCAGATAACGACTGGCTATTCA
*GATA*-*2* −4.6 kb	GAGATGAGCTAATCCCGCCGTA
	AAGGCTGTATTTTTCCAGGCT
*NECDIN* promoter	GAAGAGCTCCTGGACGCAGA
	TGCAAAGTTAGGGTCGCTCAG

### Western blot analysis

Whole cell extracts were prepared by boiling cells for 10 min in SDS sample buffer [25 mM Tris (pH 6.8), 2 % β-mercaptoethanol, 3 % SDS, 0.1 % bromophenol blue, 5 % glycerol] at 1 × 10^7^ cells/mL. Extracts from 1 to 2 × 10^5^ cells were resolved by SDS-PAGE and transferred to Hybond-P (GE Healthcare). The proteins were measured by semi-quantitatively with ECL-Plus (GE Healthcare) and CL-X Posure^TM^ Film (Thermo Scientific).

### Antibodies

Antibodies to GATA-2 (H-116) and Actin (I-19) were obtained from Santa Cruz Biotechnology. Control rabbit IgG was obtained from Abcam. Phycoerythrin (PE)-labeled human CD29, PE-labeled human CD34, fluorescein isothiocyanate (FITC)-labeled mouse/human CD44, FITC-labeled human CD45, FITC-labeled human CD90 antibodies were purchased from BD Biosciences.

The anti-GATA-2 antibody (H-116) recognizes amino acid residues 120–235 of human GATA-2.

### Flow cytometry (FACS)

The cells collected from culture were washed twice with phosphate-buffered saline (PBS; Sigma). The cells were then incubated with PE- and FITC-labeled antibodies, washed twice with PBS, and analyzed using FACSAria II (Beckton, Dickinson). The collected data were processed with FlowJo software (http://www.flowjo.com/).

### Establishment of BM-MSCs

To establish BM-MSCs, bone marrow mononuclear cells from the patient were cultured in DMEM (Life Technologies) supplemented with 20 % fetal bovine serum (Life Technologies), 10 ng/mL basic fibroblast growth factor (PeproTech), 10 mM HEPES (Life Technologies), and 100 μg/mL penicillin/streptomycin, as previously described [9]. To induce differentiation into osteroblasts and adipocytes, the hMSC Mesenchymal Stem Cell Differentiation Medium (Lonza) for osteogenic and adipogenic, respectively, was used. Osteogenic cell layers were positive for Alkaline Phosphatase staining, and typical adipocytes contained oil drops that were stained with Oil Red O.

### Genome analysis

**Fig. 1:**
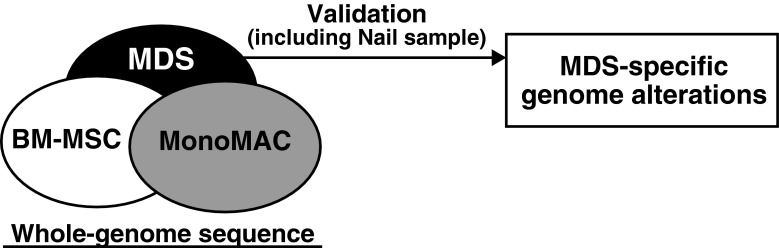
Study design. Whole-genome sequencing conducted with MonoMAC, MDS and BM-MSCs samples to identify MDS-specific genome alterations. Sanger sequence-based validation analysis was conducted on all samples, including a nail sample

To identify MDS-specific genome alterations, whole-genome sequencing was conducted on MonoMAC, MDS and BM-MSCs (Fig. 1). Genomic DNA was extracted with the DNeasy Blood & Tissue Kit (QIAGEN) or ISOHAIR (NIPPON GENE). For whole-genome sequencing, the DNA samples were amplified with the REPLI-g Midi Kit (QIAGEN). Sequencing libraries were prepared from 1 μg of the amplified DNA according to the TruSeq DNA Sample Prep Guide (Illumina). The libraries were sequenced on an Illumina HiSeq 2000 with HiSeq control software (HCS) version 1.5 and Real-Time Analysis (RTA) software version 1.13. After sequencing, reads were mapped to the human reference genome (GRCh37/hg19) with decoy sequences (hs37d5) using BWA [10] with the default options. Then, variant calling was conducted using the GATK Unified Genotyper [11].

Sanger sequence-based validation analysis was conducted with all samples, including the nail sample, using an ABI 3730xl DNA analyzer and the ABI BigDye Terminator Cycle Sequencing Kit (Applied Biosystems). The validation analyses were conducted based on the DNA samples without amplification.

## Results and discussion

### Case report

In September 2010, a 35-year-old man was referred to our department due to pancytopenia and emergence of myeloblasts in the peripheral blood. Peripheral blood count revealed a white blood cell count of 1.2 × 10^9^/L, with 79 % neutrophils, 16 % lymphocytes, 1 % eosinophils, 1 % atypical lymphocytes, and 3 % myeloblasts. The hemoglobin level was 9.8 g/dl, and the platelet count was 27 × 10^9^/L. Bone marrow analysis indicated a total nucleated cell count of 2.2 × 10^10^/L, with dysplastic morphological changes in all lineages. The bone marrow contained 11 % leukemic blasts, all positive for CD7, CD13, CD33, CD34, and HLA-DR. Cytogenetic analysis indicated the presence of trisomy 8. The patient was diagnosed with MDS, classified into the refractory anemia with excess blasts (RAEB-2), and had “Very high” risk according to the criteria of revised international prognostic index (IPSS-R) [12]. In January 2011, the case rapidly developed into AML, and received hematopoietic stem cell transplantation from a matched unrelated donor with reduced intensity conditioning regimen.

Since the age of 16 years, he had been treated for nontuberculous mycobacterial infection, cryptococcal meningitis and recurrent cutaneous human papilloma virus infection. For this reason, he was initially diagnosed with primary immunodeficiency. There was no family history of increased susceptibility to infection, or onset of MDS/AML. Based on our clinical findings and the family history, we suspected that the patient might have sporadic MonoMAC syndrome.

### Identification of a germline GATA-2 mutation

**Fig. 2:**
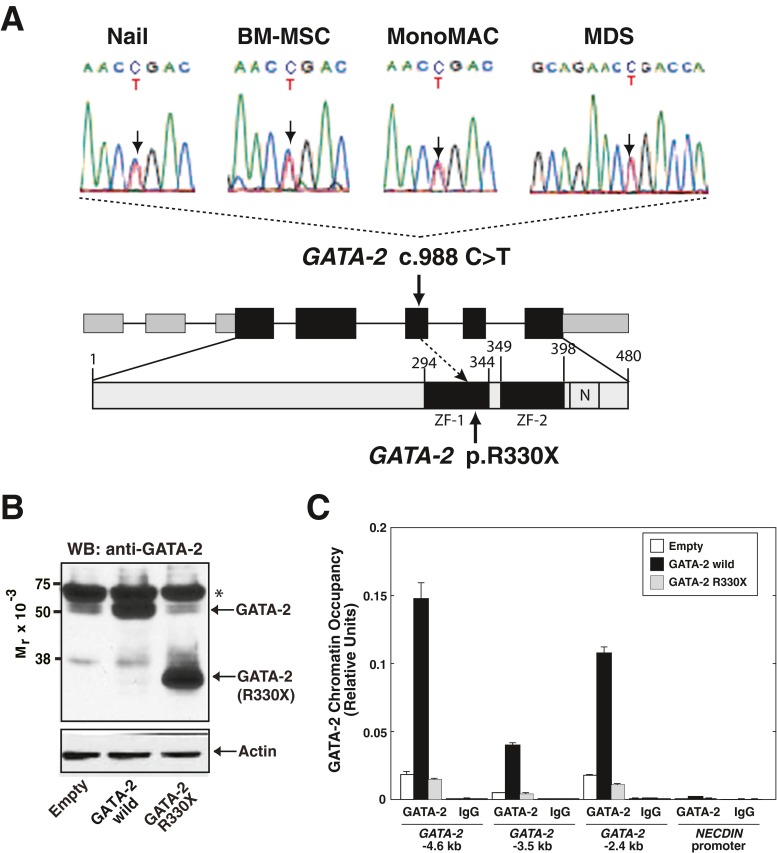
Identification of a germline heterozygous GATA-2 mutation. **a** Heterozygous germline GATA-2 mutation was confirmed with the nail, BM-MSC, MonoMAC and MDS samples: *GATA*-*2* c. 988 C > T, p. R330X. ZF, zinc finger. Numbering relative to adenine in the ATG start codon of *GATA*-*2* (GenBank NM_001145661.1) and the first methionine (GenBank NP_116027.2) [3]. **b** Western blot analysis to detect wild-type and mutated GATA-2 in K562 cells. Actin was used as a loading control. GATA-2 R330X was detected by the anti-GATA-2 antibody (H-116), as it recognizes amino acid residues 120–235 of human GATA-2. Asterisk, cross-reactive band. **c** Quantitative ChIP analysis to examine GATA-2 occupancy in wild-type and mutated GATA-2-overexpressing K562 cells (*n* = 3, mean ± SD). *NECDIN* promoter was included as a negative control

Sanger sequencing for *GATA*-*2* cDNA revealed a 988 C > T heterozygous mutation (Fig. 2a). We confirmed that the mutation was germline because it involved the nail sample (Fig. 2a). This mutation resulted in the generation of a premature stop codon at Arg330, located in the N-terminal zinc finger domain (Fig. 2a). As GATA-2 DNA binding activity is mediated through the C-terminal zinc finger domain [1], the mutated GATA-2 is predicted to be defective in DNA binding. To test the hypothesis, we overexpressed wild-type or mutated (R330X) GATA-2 in K562 cells (Fig. 2b). Quantitative ChIP analysis was conducted with anti-GATA-2 antibody at endogenous loci (GATA-2 −4.6, −3.5 and −2.4 kb), which were selected from GATA-2 ChIP-seq analysis based on K562 cells [8]. As shown in Fig. 2c, GATA-2 chromatin occupancy was obviously increased by overexpression of wild-type GATA-2. However, although the antibody could recognize GATA-2 330X (Fig. 2b), the levels of GATA-2 chromatin occupancy in GATA-2 R330X-overexpressing K562 cells were similar to the control cells. Thus, we consider that the heterozygous GATA-2 R330X mutation is a loss-of-function mutation.

### Generation and characterization of BM-MSCs

We established BM-MSCs from BM mononuclear cells from the patient, and used for the reference control for whole-genome analysis. Immunophenotypic analysis confirmed that the BM-MSCs expressed typical markers, i.e., CD29, CD44, CD90, and CD105, but not CD14, CD34, and CD45 (Supplementary Fig. 1a) [13]. Furthermore, the established BM-MSCs had the capacity to differentiate into adipocytes and osteoblasts (Supplementary Fig. 1b). Sanger sequencing also confirmed that the cells harbored the identical *GATA*-*2* mutation (Fig. 2a).

### Whole-genome sequencing identified mutations in EZH2, HECW2, and GATA-1

To elucidate the secondary genetic changes associated with the evolution to MDS/AML, we performed whole-genome sequencing with DNA samples from nail, MonoMAC, MDS, and BM-MSCs (Fig. 1). However, the data from the nail sample were excluded from the analysis due to a low mapping rate of the sequence on the human genome.

First, we focused on the MDS-specific genome deletion. GATA-2 plays a crucial role in the proliferation of hematopoietic stem cells (HSCs) [1, 14]. Thus, it is possible that *GATA*-*2* haploinsufficient HSCs could have a reduced proliferative activity. Therefore, secondary deletions involving oncogenic genes, such as a tumor suppressor gene, may promote the evolution to MDS/AML. In support of this hypothesis, a recent study revealed that a heterozygous 3-kb deletion, removing exons 7–9 of *TP53* gene, was associated with the onset of therapy-related AML through whole-genome sequencing [15]. To identify the MDS-specific gene deletion, we used several SV callers, including BreakDancer, Pindel, and CNVnator, as described previously [16]. However, we failed to identify the candidate genomic deletion that may contribute to the evolution into MDS.

**Fig. 3:**
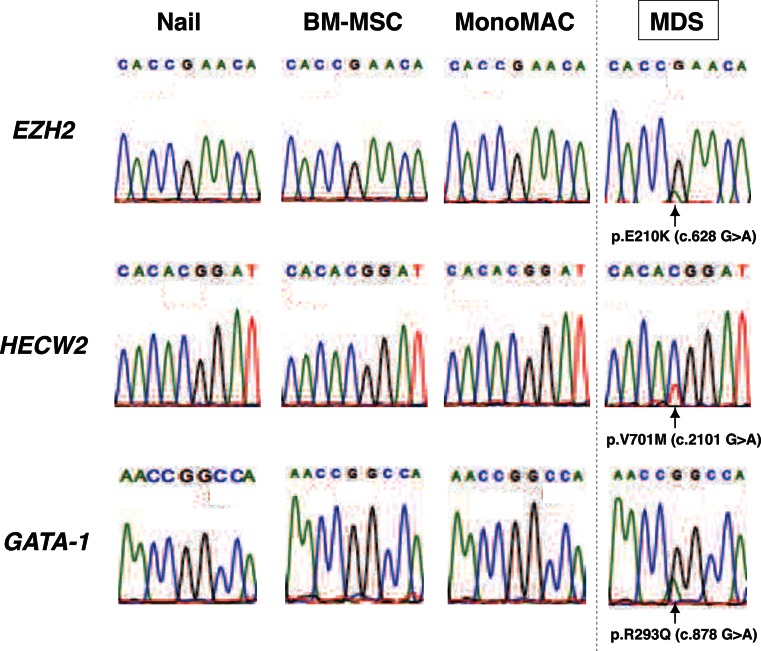
Validation analysis for MDS-specific point mutations. Validation analysis of the MDS-specific point mutations. Sanger sequence conducted to validate point mutations identified by whole-genome sequencing. For the *HECW2* mutation, the nucleotide substitution was described as opposite strand (G > A to C > T)

Our next strategy was to sort out the point mutations, using the GATK Unified Genotyper [11]. They were observed in the MDS sample, but not in MonoMAC or BM-MSCs. A total of 280 MDS-specific nonsynonymous single nucleotide variants (nsSNVs) were identified, which were subsequently narrowed down based on the single nucleotide polymorphism (SNP) database, the functional missense database and NCBI information (http://www.ncbi.nlm.nih.gov; Table 2). Finally, we identified three candidate mutations, namely EZH2 (Enhancer of zeste homolog 2, Drosophila), HECW2 (HECT, C2 and WW domain containing E3 ubiquitin protein ligase 2), and GATA-1 (GATA-binding protein 1; Table 2, Fig. 3). Sanger sequence-based validation analysis confirmed that all three mutations were observed only in the MDS sample (Fig. 3). As expected, the peak height of the mutated signal was lower than that of the wild-type signal, presumably reflecting the frequency of leukemic blasts (11 %).

**Table 2:** Point mutations identified in MDS sample

Functional missense database								
Name	Chr.	Depth	SIFT	PolyPhen2	LJB_PhyloP	LJB MutationTaster	LJB_LRT	AA Change
EZH2	7	49	0.06	0.926	0.9839	0.9995	1.0	E210K
HECW2	2	61	0.06	0.002	0.03258	0.005	0.0318	V701M
GATA-1	X	21	0	0.944	0.99824	0.9999	1.0	R293Q

Three mutations were not identified in SNP database (dbSNP130 and 1000G_ALL)

*Chr* chromosome, *SIFT* sorting intolerant from tolerant, *PolyPhen2* polymorphism phenotyping v2, *LJB_PhyloP* pathogenicity score from dbNSFP, *LJB_MutationTaster* pathogenicity probability score from dbNSFP, *LJB_LRT* pathogenicity probability score from dbNSFP, *AA* amino acid.

**Fig. 4:**
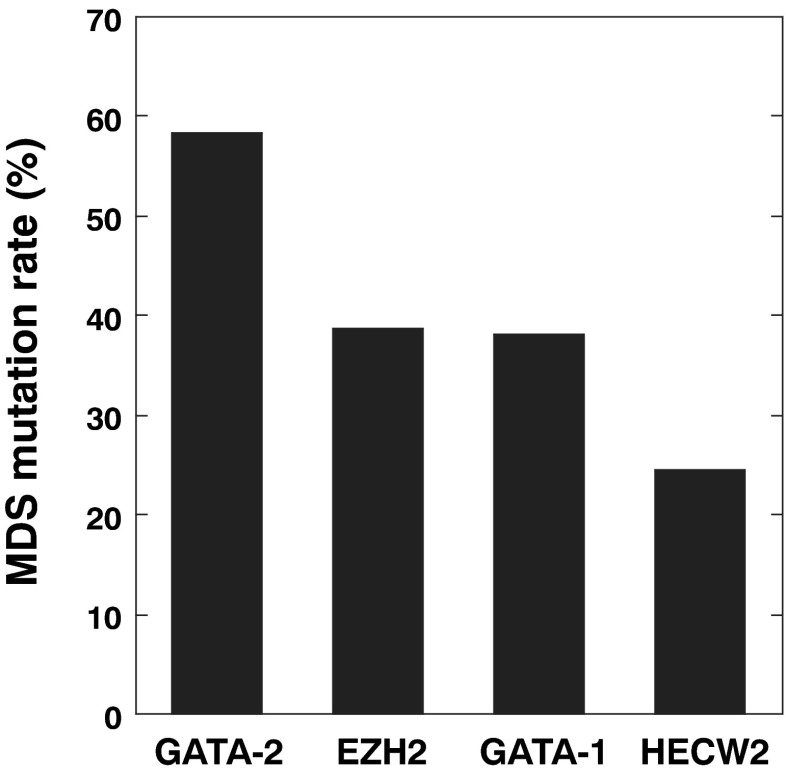
Quantification of the mutation load for EZH2, GATA-1 and HECW2. Percentage of the mutated reads per the total number of reads (both wild-type and mutated) in MDS sample was calculated for GATA-2 (c. 988 C > T), EZH2 (c. 628 G > A), GATA-1 (c. 878 G > A) and HECW2 (c. 2101 G > A)

EZH2 is a member of the Polycomb group, which is involved in the maintenance of the transcriptional repressive state of genes by trimethylation of histone H3 at lysine 27 [17]. It is well established that EZH2 loss-of-function mutations are frequently identified in MDS [18]. Amino acid position at 210 (Glu) is located at SWI3-ADA2-N-CoR-TFIIIB (SANT) domain that has been shown to interact both with histone tails and with other proteins [19]. Whereas gain-of-function mutations of EZH2 have also been attributable to pathogenesis of lymphoma, the mutations were observed at the C-terminal catalytic SET domain (Y641 and Y677) [20]. Thus, we assume that the EZH2 E210K mutation could be loss-of-function mutation. HECW2 is predicted to be ubiquitin ligase that degrades ATR (ataxia-telangiectasia-mutated-and-Rad3-related) kinase [21]. However, its role in hematopoiesis is unknown. Finally, GATA-1 is a member of the GATA transcription factors promoting erythrocyte, megakaryocyte, mast cell, and eosinophil development [1]. Amino acid position at 293 (Arg) is located in the C-terminal zinc finger domain, which is important for the recognition of the (A/T)GATA(A/G) motif, and to confer its transcriptional activity [1]. Noticeably, a recent report suggests that defective GATA-1 function results in the dyserythropoiesis, which is characteristic of MDS [22]. Based on the functional missense database, the mutations of both E210K on EZH2 and R293Q on GATA-1 may have significant effect on their endogenous functions (Table 2). In support of the hypothesis, mutation frequencies for EZH2 (38.8 %) and GATA-1 (38.1 %) were higher than HECW2 (24.6 %; Fig. 4). Thus, at least two secondary mutations in EZH2 and GATA-1 may have contributed to the early stage of clonal evolution into MDS in the present case. Future investigations are needed to determine the possible involvement of HECW2 mutation.

In conclusion, we conducted whole-genome sequencing with samples from a patient with germline GATA-2 mutation evolving immunodeficiency and MDS/AML. The new mutations we identified in *EZH2*, *HECW2* and *GATA*-*1* appear to be important secondary events leading to the development of MDS/AML of this patient.

## Electronic supplementary material

Below is the link to the electronic supplementary material.

Supplementary Fig. 1 Phenotyping of the patient-derived BM-MSCs. **a** Human BM-MSCs expressed cell-surface antigens, characteristic of BM-MSCs. **b** Differentiation of the BM-MSCs. Osteogenic cell layer was positive for alkaline phosphatase staining. Typical adipocytes contained oil drops that were stained with Oil Red O (EPS 5107 kb)

High Resulotion image (GIF 254 kb)
