# Supplementary figures and images for: Identification of acquired mutations by whole-genome sequencing in GATA-2 deficiency evolving into myelodysplasia and acute leukemia
Source: Ann Hematol. 2014 Apr 30;93(9):1515–22. doi: 10.1007/s00277-014-2090-4 (PMC4119934; doi:10.1007/s00277-014-2090-4)

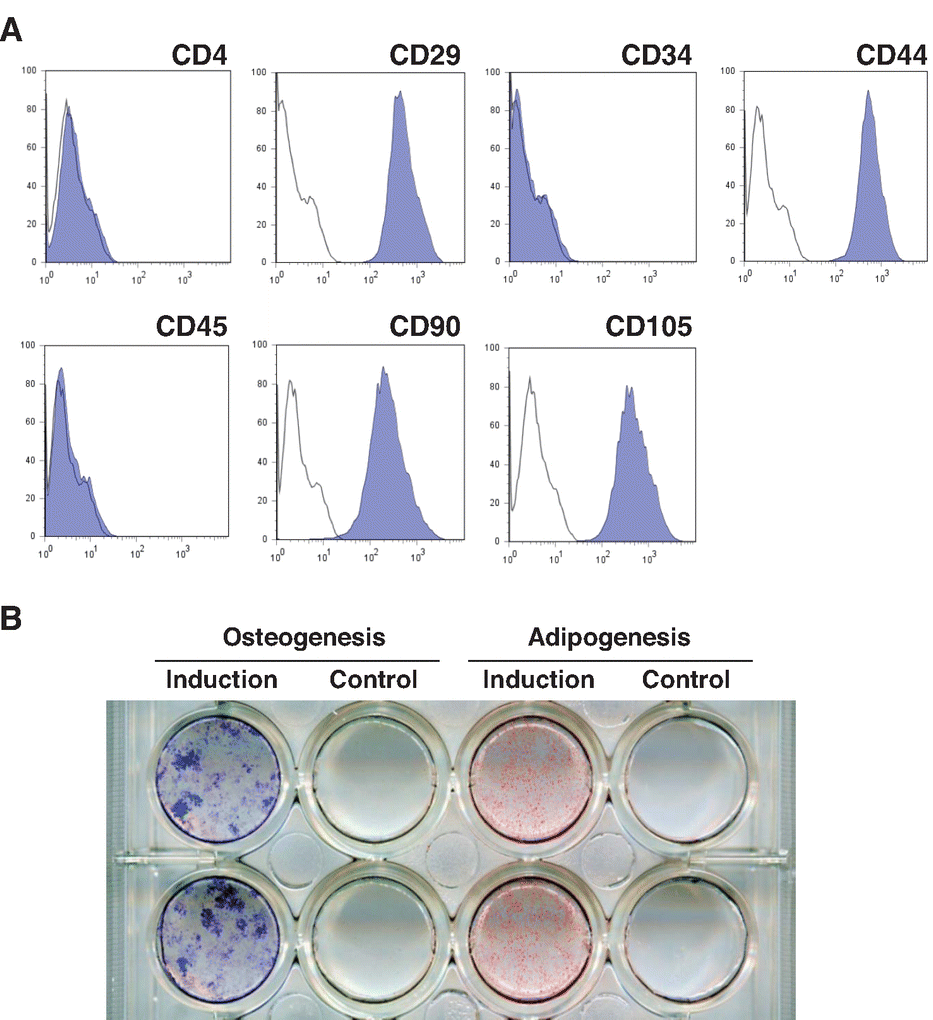

Supplement: Supplementary file 2 — High Resulotion image (GIF 254 kb) [file 277_2014_2090_Fig5_ESM.gif]
